# Advancements in the Diagnosis and Treatment of Eating Disorders in Children and Adolescents: Challenges, Progress, and Future Directions

**DOI:** 10.3390/nu17101744

**Published:** 2025-05-21

**Authors:** Omer Horovitz

**Affiliations:** 1 The Physiology & Behaviour Laboratory, Tel-Hai Academic College, 9977 North District, Qiryat Shemona 1220800, Israel; omerho1@telhai.ac.il; 2 Psychology Department, Tel-Hai Academic College, 9977 North District, Qiryat Shemona 1220800, Israel

**Keywords:** eating disorders, children, adolescents, treatment, prevention

## Abstract

Eating disorders (EDs) in children and adolescents pose significant diagnostic and therapeutic challenges due to their early onset, developmental complexity, and frequent psychiatric comorbidities. This narrative review identifies key clinical and systemic challenges, including difficulties in early detection, overlapping symptomatology, limited pharmacological options, and unequal access to specialized care. Recent progress includes revisions in diagnostic criteria (e.g., DSM-5 and ICD-11), advancements in psychometric assessment tools tailored for pediatric populations, and increasing evidence supporting psychotherapeutic interventions such as cognitive behavioral therapy, family-based therapy, and digital health approaches. Future directions involve long-term outcome studies on treatment efficacy, developing culturally sensitive and personalized care models, and implementing integrated multidisciplinary treatment frameworks. By synthesizing empirical literature from 2018 to 2024, this review underscores the urgent need for developmentally informed, evidence-based strategies to enhance the early detection, treatment, and recovery outcomes for young individuals affected by EDs.

## 1. Introduction

This review aims to update and synthesize current evidence regarding diagnosing and treating eating disorders (EDs) in pediatric populations, in light of recent changes in diagnostic classifications and the rise of digital treatment modalities. Given the evolving clinical landscape, this paper also seeks to highlight emerging challenges and propose future research directions. This conclusion is based on a synthesis of recent findings supporting the efficacy of integrated multidisciplinary care models, which have been associated with improved weight restoration, normalization of eating behaviors, and enhanced psychological well-being in pediatric populations [1,2,3,4,5].

## 2. Most Common Eating Disorders in Children and Adolescents

EDs in children and adolescents encompass a range of conditions, with anorexia nervosa (AN), bulimia nervosa (BN), binge-eating disorder (BED), and avoidant/restrictive food intake disorder (ARFID) being the most prevalent [6,7,8]. Although each disorder has distinct features, they share overlapping symptoms, making clinical diagnosis and differentiation complex, especially in younger populations [9]. In children and adolescents, diagnostic challenges often stem from limited insight into body image concerns and difficulty articulating distress, while in adolescents, symptom presentation may be more similar to that seen in adults, yet still influenced by ongoing cognitive and emotional development [10]. The clinical features of these eating disorders involve not only disordered eating behaviors but also a range of psychological and physical manifestations that impact the adolescent’s overall health and well-being [11].

### 2.1. Anorexia Nervosa (AN)

Anorexia nervosa (AN) is one of the most severe and well-studied EDs, characterized by an intense fear of gaining weight, a distorted body image, and persistent food restriction, leading to significantly low body weight [12]. In children and adolescents, AN can present with an overwhelming preoccupation with food, weight, and body shape, often leading to physical and psychological distress [13]. This disorder often involves extreme dietary restrictions, excessive exercise, and refusal to maintain a healthy weight [14]. Adolescents with AN often struggle to recognize or articulate their distorted body image, which can be compounded by developmental factors such as puberty and peer influence [15]. Additionally, younger children with AN may not express explicit body dissatisfaction but may instead show food-related anxiety or obsessive behaviors around meal times, which can complicate early detection [16].

Early detection and intervention are critical, as untreated AN can lead to long-term physical complications, including stunted growth, osteoporosis, hypothalamic amenorrhea, and cardiovascular issues [17,18]. Endocrine disruptions, such as suppression of the hypothalamic–pituitary–gonadal (HPG) axis, are particularly concerning as they can delay puberty and impair reproductive health in the long term [19]. Given the severe physical and psychological consequences of AN, early intervention and comprehensive care are essential to address the complex nature of the disorder and prevent long-term health complications.

### 2.2. Bulimia Nervosa (BN)

Bulimia nervosa (BN) is marked by recurrent binge-eating episodes followed by compensatory behaviours such as vomiting, excessive exercise, or laxative misuse [20]. Adolescents with BN may experience intense feelings of shame and guilt after binge episodes, which often lead to further compensatory behaviors [21]. Various psychosocial stressors, such as the challenges of puberty, peer pressure, and societal ideals of beauty, can trigger the development of BN in adolescents [22].

Adolescents with BN may exhibit more significant emotional dysregulation, impulsivity, and difficulty managing distress compared to those with AN [9,21,23]. Research suggests that BN is strongly associated with deficits in self-regulation, leading to binge episodes as a maladaptive coping mechanism [24]. While BN is often more visible in its presentation due to compensatory behaviors, it shares many of the same psychological underpinnings as anorexia nervosa, including issues with self-esteem, emotional regulation, and a distorted body image [6,7,9]. The impact of BN can be profound, leading to electrolyte imbalances, gastrointestinal issues, and potential damage to the teeth and esophagus due to frequent vomiting [25]. If left untreated, severe BN can lead to life-threatening complications, including cardiac arrhythmias due to hypokalemia [26].

In conclusion, BN represents a complex and profoundly challenging disorder, particularly among adolescents, who are particularly susceptible to the pressures of body image and societal expectations. The hidden nature of the condition, combined with its psychological and physical consequences, highlights the importance of early detection, compassionate care, and comprehensive treatment strategies to address both the underlying emotional struggles and the physical health risks associated with BN.

### 2.3. Binge-Eating Disorder (BED)

Binge-eating disorder (BED) shares similarities with bulimia nervosa, particularly the presence of binge episodes, but lacks compensatory behaviors such as vomiting or excessive exercise [27]. Adolescents with BED often consume large quantities of food in a short period, accompanied by distress and a loss of control [28]. Unlike AN or BN, BED is not primarily driven by a fear of weight gain but rather by a dysregulated reward system, emotional distress, and impaired impulse control [27,28,29]. As a result, adolescents with BED may experience significant emotional distress and may also struggle with weight gain due to the frequency and magnitude of the binge episodes [30].

This disorder is often associated with comorbid conditions such as depression, anxiety, and low self-esteem, and it can significantly impair a young person’s psychological well-being [31]. Neurobiological research indicates that individuals with BED may exhibit heightened reward sensitivity to food cues, contributing to compulsive overeating [32]. Furthermore, studies have shown that adolescents with BED are at increased risk of developing obesity, metabolic syndrome, and other long-term health problems [33]. Early intervention is crucial for managing BED and addressing the underlying emotional and psychological issues that often drive these binge episodes [34]. In conclusion, BED is a complex eating disorder that, while distinct from AN and BN, presents significant emotional and physical challenges, particularly in adolescents. The disorder’s strong ties to emotional distress, coupled with the risk of long-term health complications, highlight the importance of early identification and intervention to help adolescents manage both the psychological triggers and physical outcomes associated with BED.

### 2.4. Avoidant/Restrictive Food Intake Disorder (ARFID)

Avoidant/Restrictive Food Intake Disorder (ARFID) is a relatively recent addition to the diagnostic categories of eating disorders and is increasingly recognized in pediatric populations [35]. ARFID is marked by extreme food avoidance and a lack of interest in eating, often leading to nutritional deficiencies and stunted growth [35,36]. Unlike anorexia nervosa, ARFID is not motivated by a desire to lose weight or a distorted body image but rather by sensory sensitivities, a heightened aversion to certain food textures, colors, or smells, or a lack of interest in food altogether [37].

Children with ARFID may refuse to eat a wide variety of foods, which can result in malnutrition and developmental delays. Unlike AN or BN, children with ARFID often express fear of choking or vomiting, which can lead to a conditioned avoidance of food. This condition can be particularly challenging to diagnose because it often overlaps with other issues, such as autism spectrum disorders or anxiety [38]. The absence of compensatory behaviors, such as purging or over-exercising, distinguishes ARFID from other eating disorders like BN or AN [39]. As with other eating disorders, ARFID often co-occurs with comorbid psychiatric conditions, including anxiety, depression, and obsessive–compulsive tendencies [40], further complicating the clinical picture and necessitating a multidisciplinary treatment approach.

In conclusion, ARFID is an often-overlooked eating disorder that poses distinct challenges for diagnosis and treatment, particularly in children and adolescents. Unlike other eating disorders, ARFID is caused by sensory sensitivities or a lack of interest in food, not by worries about body image or weight. Because it can lead to serious nutritional problems and developmental delays, it is essential to recognize it early and take a complete, team-based approach to treat the physical and emotional aspects.

### 2.5. Comorbidities and Psychological Impact

In children and adolescents, eating disorders are often accompanied by comorbid mental health issues such as depression, anxiety, obsessive–compulsive disorder (OCD), and even attention-deficit/hyperactivity disorder (ADHD) [41]. There is a bidirectional relationship between EDs and these conditions, as anxiety and mood disorders may precede or develop as a consequence of disordered eating behaviors. These comorbidities not only complicate the diagnosis but also hinder treatment, as addressing the ED may not resolve the underlying psychological issues. Additionally, the social pressures faced by adolescents, including concerns about body image, peer acceptance, and societal standards of beauty, often exacerbate the severity and persistence of these disorders [42]. Adolescents with eating disorders are also at heightened risk for social isolation, academic difficulties, and strained family relationships, making the treatment of EDs an intricate and multifactorial challenge [43,44].

The clinical features of EDs in children and adolescents are diverse and multifaceted. Recognizing these symptoms and understanding the psychological and developmental factors behind them are key to accurate diagnosis and effective treatment. Given the overlapping nature of these disorders and the frequent presence of comorbid mental health conditions, early identification and intervention are crucial in mitigating the long-term impact of eating disorders on the physical, psychological, and social development of young people. For a summary of key features, psychological factors, physical consequences, and diagnostic challenges of EDs in children and adolescents, see Table 1.

**Table 1:** Clinical features of eating disorders in children and adolescents.

Eating Disorder	Key Features	Psychological Factors	Physical Consequences	Diagnostic Challenge
Anorexia Nervosa (AN)	Intense fear of weight gain, distorted body image, food restriction, excessive exercise [12,13,14]	Perfectionism, anxiety, peer influence, limited insight into body image concerns [15,16]	Stunted growth, osteoporosis, hypothalamic amenorrhea, cardiovascular issues, delayed puberty [17,18,19]	Younger children may not express body dissatisfaction explicitly; symptoms can be masked by developmental factors [16]
Bulimia Nervosa (BN)	Binge eating followed by compensatory behaviors (vomiting, excessive exercise, laxative use), intense shame/guilt [20,21]	Emotional dysregulation, impulsivity, low self-esteem, peer pressure, societal ideals [9,22,23,24]	Electrolyte imbalances, gastrointestinal issues, dental erosion, cardiac arrhythmias [25,26]	Symptoms may be hidden due to secrecy and shame; impulsive behaviors complicate diagnosis [9]
Binge-Eating Disorder (BED)	Recurrent binge episodes without compensatory behaviors, distress and loss of control over eating [27,28]	Emotional distress, reward-system dysregulation, low self-esteem, depression, anxiety [29,30,31]	Increased risk of obesity, metabolic syndrome, long-term health issues [32,33]	Often misdiagnosed as simple overeating; emotional factors may be overlooked [34]
Avoidant/Restrictive Food Intake Disorder (ARFID)	Extreme food avoidance, nutritional deficiencies, lack of interest in food, fear of choking or vomiting [35,36,37]	Sensory sensitivities, anxiety, OCD-like tendencies, autism spectrum association [38,39,40]	Malnutrition, developmental delays, failure to thrive [39]	Often overlaps with other conditions like ASD; lack of weight/shape concerns differentiates it from AN/BN [39]
Comorbidities and Psychological Impact	Co-occurrence with depression, anxiety, OCD, ADHD; heightened risk for social isolation and academic difficulties [41,42,43]	Anxiety and mood disorders may precede or result from EDs, bidirectional relationship with psychiatric conditions [41,42]	Increased vulnerability to long-term mental health issues [44]	Underlying psychological conditions may mask or exacerbate ED symptoms, complicating treatment [43,44]

### 2.6. Nosological Updates in Diagnostic Systems

Recent revisions to psychiatric diagnostic systems have aimed to improve developmental sensitivity in the classification of eating disorders. Compared to DSM-IV, DSM-5 removes the amenorrhea criterion and lowers the frequency thresholds for bulimia nervosa and binge-eating disorder, facilitating earlier diagnosis and broader clinical applicability. ICD-11, unlike ICD-10, removes strict weight thresholds and emphasizes developmental and cultural considerations in its diagnostic guidance [45,46,47]. The DSM-5 introduced ARFID as a distinct diagnosis, replacing the narrower “Feeding Disorder of Infancy or Early Childhood” from DSM-IV. ARFID broadens the clinical scope to include children and adolescents who exhibit restrictive eating not driven by body image concerns but who nonetheless experience significant nutritional, growth, or psychosocial impairments [45]. In addition, DSM-5 revised the diagnostic thresholds for AN and BN, such as removing the amenorrhea criterion and lowering the frequency requirements for binge/purge behaviors, thereby facilitating earlier identification and intervention [46]. Similarly, ICD-11 reflects a shift toward earlier and more flexible diagnosis by removing strict weight thresholds and incorporating developmental and cultural considerations in the diagnostic guidance for EDs [47]. These nosological updates better account for the heterogeneity of pediatric eating disorder presentations and support a more inclusive and developmentally appropriate clinical framework.

## 3. Assessment Strategies

This narrative review is based on a comprehensive synthesis of recent literature published between 2018 and 2024. Relevant articles were identified using databases such as PubMed, PsycINFO, and Scopus, using keywords including ‘eating disorders’, ‘children’, ‘adolescents’, ‘treatment’, and ‘diagnosis.’ Preference was given to high-quality reviews, clinical trials, and meta-analyses involving pediatric populations. As this is a narrative review, no formal quality assessment tool was applied. Studies were selected based on clinical relevance, methodological rigor, and applicability to pediatric populations. As this is a narrative review, no formal quality appraisal tool was applied. Findings were selected based on relevance, methodological robustness, and applicability to pediatric populations, but the heterogeneity in study designs and sample characteristics limits direct comparisons. Future systematic reviews using standardized quality assessment criteria are needed to more accurately evaluate the strength of evidence across intervention types and populations.

Given the spectrum of EDs in children and adolescents and the variability in their presentation, a comprehensive, multidimensional assessment is crucial for accurate diagnosis and effective treatment. EDs present unique challenges in younger populations, as symptoms can be subtle, overlap with other conditions, and be influenced by developmental factors. As a result, the assessment process must be thorough and tailored to the individual, incorporating psychological, medical, and family evaluations to capture the disorder’s full scope and impact on the adolescent’s health.

### 3.1. Psychological Assessment

Psychological evaluation is a key component of the assessment process for children and adolescents with eating disorders. Tools such as the Eating Disorder Examination (EDE) [48], Eating Disorder Inventory (EDI) [49], and the Child Eating Disorder Examination (ChEDE) [50] are commonly employed to assess the core features of EDs, eating behaviors, cognitive distortions, and emotional factors contributing to the disorder. These instruments provide valuable insights into the individual’s attitudes toward food, body image, weight, and the psychological distress associated with these concerns.

Using structured interviews and age-appropriate questionnaires is crucial for adolescents, particularly those who struggle to articulate their emotions or understand their behaviors [51]. Research has shown that using tools tailored to developmental stages, such as the ChEDE, helps clinicians more accurately capture the symptoms of eating disorders in younger individuals [51]. For younger children, assessment often involves direct observations and structured interviews with the child and their caregivers to gather a comprehensive view of eating habits, emotional states, and family dynamics [52]. This holistic approach enables clinicians to gain a more nuanced understanding of the contributing factors and develop targeted interventions.

For example, the Youth Eating Disorder Examination Questionnaire (YEDE-Q) is a brief, self-report tool that is ideal for use in school settings or for large-scale screenings. At the same time, the ChEDE offers more detailed clinical insights through structured interviews. The Questionnaire on Eating and Weight Patterns–Adolescent Version (QEWP-A) is particularly useful for identifying binge-eating patterns, and the Pediatric Eating Disorder Examination Questionnaire (PEDE-Q) may be better suited for caregiver reports in pediatric settings where the child is unable to self-report reliably.

The YEDE-Q and QEWP-A demonstrate strong internal consistency and construct validity in adolescent populations [53,54]. The YEDE-Q, a self-report adaptation of the ChEDE, has shown significant agreement on all four subscales, global score, and the assessment of objective bulimic episodes, confirming its reliability in evaluating eating-related pathology among overweight adolescents [55,56]. Similarly, the QEWP-A has demonstrated adequate test–retest reliability and has been effectively used to identify adolescent binge-eating behaviors, with studies reporting significant stability over three weeks [54,57].

These tools are particularly valuable in outpatient or school-based settings where time constraints or developmental limitations may reduce the feasibility of clinician-administered interviews. The PEDE-Q also shows promise as a developmentally appropriate tool, though further validation is required in larger clinical samples [58]. Continued psychometric refinement and population-based validation will be essential for broader clinical and community implementation.

In conclusion, psychological evaluation plays a vital role in the comprehensive assessment and treatment of eating disorders in children and adolescents. By utilizing age-appropriate tools and involving the child and their caregivers, clinicians can better understand the complex interplay between eating behaviors, cognitive distortions, and emotional factors. This comprehensive approach ensures more accurate diagnosis and helps inform tailored interventions that address the unique developmental and psychological needs of younger individuals facing eating disorders.

### 3.2. Medical Assessment

Given the severe and potentially life-threatening effects of eating disorders, especially AN and bulimia nervosa, a thorough medical assessment is essential. Children and adolescents with EDs often experience significant disruptions in physical health, including malnutrition, electrolyte imbalances, and delayed puberty, which may require intervention from a pediatrician, pediatric endocrinologist, or adolescent medicine specialist [25,59]. A comprehensive physical examination typically includes monitoring weight, height, and growth charts to assess deviations from expected developmental trajectories, blood tests to evaluate electrolyte imbalances, liver and kidney function, and nutritional deficiencies [60], cardiovascular assessments, as adolescents with AN may exhibit bradycardia, hypotension, and other cardiovascular complications requiring urgent intervention [61], and bone density scans, particularly for those at risk of osteoporosis due to prolonged malnutrition and hormonal disruptions [62].

Medical evaluations also provide insights into pubertal development, as eating disorders can delay or disrupt standard growth patterns. In severe cases of eating disorders, hospitalization may be necessary for intensive monitoring and stabilization. Recent research suggests that factors such as age, BMI, EDE score, social risks, and psychiatric comorbidities—including autism spectrum disorder—can predict the likelihood and duration of hospitalization in adolescents with EDs [63].

In conclusion, a comprehensive and ongoing medical assessment is critical in addressing the severe physical consequences of eating disorders. Regularly monitoring growth, cardiovascular function, and organ health is essential to identify potential medical risks promptly. In severe cases, hospitalization may be required for stabilization and intensive intervention. Understanding the interplay between physical health, psychological factors, and comorbidities is essential for optimizing treatment outcomes in these vulnerable individuals.

### 3.3. Family Assessment and Therapy

Family dynamics play a key role in the development, maintenance, and recovery of eating disorders, making family-based assessments essential for understanding environmental and relational factors contributing to the disorder. Family-based therapy (FBT), particularly the Maudsley approach, is a widely studied and frequently implemented intervention for adolescents with EDs [64].

FBT is founded on the principle that parents are the most effective agents of change in treating adolescent EDs. It empowers parents to participate actively in their child’s recovery. The initial focus is on weight restoration and behavioral change, gradually transitioning toward fostering autonomy in eating behaviors [65]. FBT has demonstrated substantial evidence in reducing symptom severity and improving family dynamics in adolescents with AN [38,66].

Beyond FBT, other family-focused therapeutic approaches may be beneficial. Emotion-focused family therapy (EFFT) integrated with FBT aims to support parents in refeeding, symptom interruption, and emotional coaching, offering a promising approach for families requiring more intensive eating disorder treatment [67]. Structural-strategic family Therapy (SFT) is an effective intervention for adolescents with mental health issues and their families, improving adolescent behaviour, family cohesion, parental competence, and parenting practices while also addressing dysfunctional family roles that may contribute to disordered eating [68]. Lastly, cognitive behavioral family interventions focus on challenging maladaptive beliefs surrounding food, weight, and self-perception [66].

Family involvement is crucial for younger children, who rely heavily on caregivers for emotional regulation and support. Effective family interventions target the eating disorder and enhance overall family functioning, communication, and emotional resilience [38]. Table 2 summarizes the comprehensive assessment strategies for eating disorders in children and adolescents.

**Table 2:** Comprehensive assessment strategies for eating disorders in children and adolescents.

Assessment Type	Key Component	Methods/Tools	Significance
Psychological Assessment	Evaluates cognitive, emotional, and behavioral aspects of EDs	- Eating Disorder Examination (EDE) [48] - Eating Disorder Inventory (EDI) [49] - Child Eating Disorder Examination (ChEDE) [50] - Structured interviews and age-appropriate questionnaires [51] - Direct observations and caregiver interviews for younger children [52]	Identifies core symptoms, eating behaviors, cognitive distortions, and emotional distress; tailored tools enhance accuracy for younger individuals [51]
Medical Assessment	Assesses physical health impacts of EDs	- Weight, height, and growth chart monitoring [13,59] - Blood tests (electrolytes, liver/kidney function, nutritional deficiencies) [60] - Cardiovascular assessments (bradycardia, hypotension) [61] - Bone density scans (osteoporosis risk) [62] - Pubertal development monitoring [13] - Hospitalization based on risk factors such as BMI, EDE score, social risks, and psychiatric comorbidities [63]	Identifies malnutrition effects, medical risks, and comorbidities; regular monitoring helps detect immediate threats to health [63]
Family Assessment and Therapy	Evaluates family dynamics and their role in EDs	- Family-Based Therapy (FBT) (e.g., Maudsley Approach) [64] - Emotion-Focused Family Therapy (EFFT) [67] - Structural–Strategic Family Therapy (SFT) [68] - Cognitive Behavioral Family Interventions [66]	Strengthens parental involvement in recovery, improves family dynamics, and enhances emotional resilience in younger individuals [66]

## 4. Intervention Approaches

Recent advances in treating eating disorders in children and adolescents emphasize a multidisciplinary approach, integrating psychotherapy, medical management, and nutritional support. While some treatment modalities are well-established, ongoing research continues to identify new strategies to improve outcomes for young patients.

### 4.1. Psychotherapy

Cognitive behavioral therapy (CBT) is widely regarded as one of the most evidence-based and effective treatments for eating disorders across age groups [66,69]. However, adaptations are necessary when working with children and adolescents. CBT for adolescent EDs often incorporates family therapy, recognizing the importance of parental involvement in supporting recovery and long-term outcomes [70]. For instance, the enhanced form of CBT (CBT-E) has been adapted to better address the needs of adolescents, placing greater emphasis on developmental considerations, such as peer influences and identity formation, while also targeting eating behaviors and underlying emotional issues like low self-esteem and body dissatisfaction [71].

Additionally, interpersonal psychotherapy (IPT) and dialectical behavior therapy (DBT) are gaining recognition for their effectiveness, particularly in cases with co-occurring emotional dysregulation, such as depression or trauma [72,73]. IPT focuses on improving interpersonal relationships and reducing emotional distress [72]. At the same time, DBT helps adolescents manage intense emotions and build coping skills, which can be particularly beneficial for those with comorbid mood disorders [73]. Other emerging approaches, such as emotion-focused therapy (EFT) and mentalization-based therapy (MBT), show promise in addressing attachment-related difficulties and emotion regulation deficits in adolescents with EDs, but further research is needed to establish their efficacy in clinical practice [67,74]. For example, enhanced CBT-E led to a 45% reduction in binge-eating episodes over 12 weeks in adolescents with BED [71]. Additionally, FBT achieved remission rates of approximately 72% at 6-month follow-up in adolescents with anorexia nervosa [64].

In conclusion, while CBT remains the gold standard for treating eating disorders, adaptations like CBT-E, which integrates family therapy, and the use of alternative approaches such as IPT and DBT, are crucial for addressing the unique emotional and interpersonal needs of adolescents. Newer interventions, such as EFT and MBT, may provide additional benefits for specific populations, highlighting the need for continued research to refine therapeutic options. These tailored therapies provide a comprehensive framework for treating the complex psychological factors underlying eating disorders in young individuals.

### 4.2. Nutritional Rehabilitation

Nutritional rehabilitation is a cornerstone of ED treatment, particularly for adolescents. It aims to restore standard eating patterns and address the physical and nutritional deficiencies caused by restrictive eating behaviors [75,76]. Pediatric dietitians and nutritionists are vital members of the treatment team. They collaborate with adolescents and their families to develop structured meal plans that meet nutritional needs and support growth and development. These plans are often tailored to the adolescent’s specific stage of development, factoring in both their immediate health needs and long-term goals.

Research highlights the crucial role of nutritional rehabilitation in the recovery process. A multidisciplinary approach, integrating nutritional support with psychological therapy, has been associated with better weight restoration, normalization of eating behaviors, and overall psychological well-being [2]. Given the multifaceted etiology of eating disorders, dietitians play a crucial role in addressing both medical and psychosocial aspects, ensuring a comprehensive, supportive treatment process. Additionally, research indicates that fostering a sense of autonomy and control over food choices within a structured framework enhances adherence to treatment and long-term recovery [77].

Early interventions incorporating preventive strategies, such as positive body image and nutrition education, have been shown to prevent the persistence of disordered eating behaviors [78]. For instance, a school-based intervention targeting body image, social media usage, and lifestyle factors significantly reduced eating disorder risk factors and enhanced protective factors, demonstrating sustained positive effects over 12 months [79]. These findings highlight the importance of integrating preventive education into public health initiatives to mitigate the onset and chronicity of eating disorders.

In conclusion, nutritional rehabilitation extends beyond physical recovery, encompassing psychological and behavioral support to address the root causes of eating disorders. When performed in a structured, supportive, and developmentally appropriate manner, it plays a pivotal role in recovery. Integrating early intervention strategies, such as body image education and media literacy, into public health programs could reduce ED risk and improve adolescent long-term outcomes.

### 4.3. Pharmacotherapy

While medications are not the first-line treatment for EDs, pharmacotherapy plays an essential role in managing adolescents with severe or chronic EDs, especially those with comorbid conditions like depression, anxiety, or OCD. Recent research on pharmacological treatments for eating disorders shows that olanzapine (an atypical antipsychotic) is effective in promoting weight gain in AN, while fluoxetine (a selective serotonin reuptake inhibitor (SSRI)) and lisdexamfetamine (a stimulant used to treat ADHD) are FDA-approved treatments for BN and BED, respectively [80]. Although SSRIs are commonly used off-label for BN and BED, ongoing research is needed to clarify their long-term efficacy and optimal dosage for adolescents.

Despite these advancements, treatment options remain limited for certain EDs, such as ARFID and other specified feeding and eating disorders (OSFED). There are no FDA-approved pharmacological treatments for ARFID, underscoring the need for further research in this area [81]. SSRIs help address the emotional and psychological distress associated with EDs, particularly in cases with anxiety and obsessive-compulsive traits [82]. Similarly, existing fluoxetine was effective in reducing the frequency of binge-eating episodes and improving overall psychological well-being in adolescents with bulimia nervosa [83,84]. Furthermore, SSRIs were found to enhance the efficacy of CBT in youth with depression and anxiety [85].

However, most randomized controlled trials (RCTs) examining the combination of pharmacological and psychological treatments for AN, BN, and BED have yielded mixed results, with few showing significant additional benefits over psychotherapy alone. Exceptions include fluoxetine, which has shown some effectiveness when combined with structured therapy for BN, and certain antiseizure medications that may reduce binge-eating episodes in BED [86]. These findings suggest that while medications can provide symptom relief for some individuals, their role in the overall treatment of EDs remains secondary to psychological and nutritional interventions.

In conclusion, while pharmacotherapy is not a primary treatment for eating disorders, it plays a crucial role in managing adolescents with severe or chronic EDs, particularly those with comorbid conditions like depression, anxiety, or OCD. Medications such as olanzapine for AN, fluoxetine for BN, and lisdexamfetamine for BED have shown efficacy. However, the overall evidence supporting the additive benefits of combining medication with psychotherapy is limited, and further research is needed to optimize pharmacological treatment strategies, particularly for under-researched disorders like ARFID. Regarding safety and tolerability, olanzapine has been associated with mild sedation and increased appetite; however, it was generally well-tolerated in adolescents with AN [80]. Similarly, fluoxetine was well-tolerated and significantly reduced binge-purge behaviors in adolescents with bulimia nervosa, showing sustained improvement across a 12-week trial [83].

As treatment approaches evolve, a more nuanced understanding of when and how to integrate pharmacotherapy into multidisciplinary ED care is essential. Integrated care models—combining psychological therapy, nutritional rehabilitation, and medical monitoring—have shown promise in improving treatment outcomes, particularly in complex cases with medical and psychiatric comorbidities [87,88]. While more longitudinal research is needed, this review supports integrative approaches as promising evolving models across pediatric ED treatment domains. Table 3 summarizes the multidisciplinary intervention approaches for eating disorders in children and adolescents.

**Table 3:** Multidisciplinary intervention approaches for eating disorders in children and adolescents.

Treatment Component	Key Points	Methods/Tools	Significance
Psychotherapy	Addresses cognitive, emotional, and behavioral factors contributing to EDs	- Cognitive Behavioral Therapy (CBT) [69] - Enhanced CBT (CBT-E) with family integration [71] - Interpersonal Psychotherapy (IPT) for emotional distress and relationships [72] - Dialectical Behavior Therapy (DBT) for emotional regulation [73] - Emerging therapies: Emotion-Focused Therapy (EFT) and Mentalization-Based Therapy (MBT) [67,74]	CBT remains the gold standard, but adaptations (CBT-E) and alternatives (IPT, DBT) enhance treatment for adolescents with comorbid emotional and interpersonal challenges [73]
Nutritional Rehabilitation	Restores eating patterns and addresses nutritional deficiencies	- Structured meal plans tailored to growth and development [75] - Multidisciplinary approach involving dietitians, psychologists, and physicians [76] - Autonomy-supportive feeding strategies [77] - Preventive education: body image, media literacy, school-based interventions [78,79]	Critical for physical and developmental recovery; prevention programs reduce ED risk factors and promote resilience [78,79]
Pharmacotherapy	Supports symptom management, particularly for comorbid conditions	- Olanzapine for weight gain in AN [80] - Fluoxetine (SSRI) for BN and BED [83,84] - Lisdexamfetamine for BED [80] - SSRIs for comorbid anxiety, depression, OCD [88,89] - Limited pharmacological options for ARFID; ongoing research needed [81]	Pharmacotherapy is adjunctive, not primary; useful in severe or treatment-resistant cases, especially with comorbidities. Efficacy is mixed and requires further RCT validation [90]

## 5. Innovative Approaches

Advances in digital health and telemedicine have significantly transformed the treatment landscape for EDs, offering innovative solutions for adolescents who face barriers to in-person care due to geographic location, financial limitations, or social stigma. Digital interventions, such as online CBT programs, virtual family therapy sessions, and mobile apps designed to track eating behaviors and mental health symptoms, have shown promising efficacy in improving treatment access and enhancing engagement with care [91]. For instance, a pilot study demonstrated that delivering the Body Image module of enhanced CBT-E in a virtual group setting was not only feasible but also well-received by adolescents with eating disorders, leading to positive feedback and significant reductions in disorder-related impairment [92]. These digital platforms enable real-time monitoring, personalized feedback, and enhanced continuity of care, effectively bridging the treatment gap for individuals in rural or underserved regions with limited access to specialized ED care.

A systematic review and meta-analysis further reinforce the growing evidence supporting digital interventions. These interventions show promise as effective treatments for eating disorders, demonstrating significant benefits in both prevention and treatment, particularly for cognitive symptoms [89]. However, the evidence base remains limited, particularly regarding relapse prevention, and further high-quality randomized controlled trials (RCTs) are required to confirm their long-term effectiveness. Nevertheless, such findings underscore the potential of digital health to overcome logistical barriers such as long travel times and limited local resources, making it a compelling alternative or adjunct to traditional face-to-face treatment.

In addition to digital interventions, inpatient and residential treatment programs, along with adolescent eating disorder day programs (DPs), are essential for treating adolescents with severe eating disorders. Inpatient programs offer intensive care, combining medical stabilization, psychological therapy, and nutritional support to provide a comprehensive, multidimensional approach to recovery [90]. Similarly, DPs have proven to be an effective alternative to inpatient care, with evidence showing sustained improvements in weight gain and psychopathology [93]. While both treatment options integrate medical care, therapy, and family involvement, DPs offer a more flexible model that can benefit adolescents who do not require complete inpatient care. However, the variability in day program models—particularly in treatment approaches and intensity—complicates direct comparisons and outcome assessments. Therefore, more controlled studies are needed to examine the impact of different therapeutic models, treatment mechanisms, and the role of family involvement, ultimately guiding the optimization of these programs for diverse patient needs.

The assessment and treatment of eating disorders in children and adolescents require a multidisciplinary, individualized approach that recognizes the complex interplay between psychological, physical, and familial factors. Early identification and intervention are critical to minimizing the long-term impact of eating disorders on the developing adolescent. Essential components such as psychological evaluations, medical assessments, and family-based interventions play pivotal roles in this comprehensive approach, helping to mitigate the damaging effects of EDs on young individuals. Early intervention strategies, particularly when they incorporate psychotherapy and nutritional rehabilitation, can significantly reduce the severity and chronicity of the disorder, improving long-term outcomes for affected adolescents [34]. Moreover, ongoing advancements in treatment modalities, including pharmacotherapy and digital health tools, have broadened the scope and availability of care options for adolescents with eating disorders. Integrating telemedicine and digital tools with traditional therapeutic approaches offers exciting possibilities for the future, enabling personalized, continuous care that enhances the chances of recovery. Future research will likely focus on optimizing digital interventions, exploring their long-term efficacy, and integrating them into traditional treatment frameworks [94].

In conclusion, the evolving landscape of eating disorder treatment, which includes digital health solutions, innovative psychotherapy models, and intensive inpatient care, provides hope for adolescents affected by EDs. With ongoing advancements in care delivery and treatment options, there is growing optimism for achieving better outcomes, improving the availability and accessibility of services, and ensuring that adolescents with eating disorders receive the comprehensive, personalized care they need for recovery. Table 4 summarizes the innovative approaches in eating disorder treatment for children and adolescents.

**Table 4:** Innovative approaches in eating disorder treatment for adolescents.

Approach	Key Points	Methods/Tools	Significance
Digital Health and Telemedicine	Expands access to treatment and enhances engagement	- Online CBT programs and virtual family therap [91] - Mobile apps for tracking eating behaviors and mental health [91] - Virtual delivery of CBT-E Body Image module [92] - Real-time monitoring and personalized feedback [91]	Improves accessibility for underserved populations; enhances continuity of care, but more research needed on long-term effectiveness and relapse prevention [89]
Day Programs (DPs) and Inpatient Care	Intensive, structured treatment for severe ED cases	- Inpatient programs for medical stabilization, therapy, and nutritional support [90] - Day Programs (DPs) as structured, flexible alternatives to inpatient care [93] - Family involvement in treatment models [93]	Have demonstrated effectiveness for some adolescents with severe presentations; DPs offer a less restrictive alternative, but program variability complicates comparisons; further research needed to guide optimization [93]
Integration of Digital and Traditional Treatment	Combines digital and in-person approaches for comprehensive care	- Hybrid models merging telemedicine with face-to-face therapy [94] - Digital tools to improve treatment accessibility and outcomes [94]	Promising for enhancing personalization and continuity of care; requires further evidence to determine optimal approaches and long-term efficacy [94]

## 6. Advances in Eating Disorders in Children and Adolescents

Eating disorders are complex psychiatric conditions that often emerge during childhood and adolescence, which are critical periods of physical, emotional, and psychological development [6]. Various risk factors contribute to their onset, including genetic predisposition, social pressures, early dieting behaviors, and perfectionistic traits. These disorders, manifesting during such a formative period, have profound implications for adolescents’ growth, self-esteem, and long-term well-being [95,96]. Diagnosing and treating eating disorders in pediatric populations is a complex tasks due to the wide variety of presentations across subtypes such as AN, BN, BED, and ARFID [7]. These disorders often share overlapping symptoms, complicating clinical differentiation. For instance, anorexia nervosa, often marked by extreme weight restriction and distorted body image, is commonly accompanied by anxiety, depression, and obsessive-compulsive behaviors [97,98,99].

In treatment, the field has made significant strides in developing more effective interventions for pediatric populations. CBT, particularly its adapted forms, such as CBT-E, is regarded highly supported and frequently recommended approach for treating eating disorders in adolescents [71]. CBT-E, tailored to the specific developmental needs of adolescents, leads to significant improvements in eating disorder psychopathology and can be particularly beneficial in reducing the frequency of binge episodes and restrictive eating behaviors [69]. Furthermore, the inclusion of FBT, which involves parents in managing the disorder through structured support and meal management, has shown robust evidence of effectiveness for anorexia nervosa in adolescents, with multiple randomized controlled trials indicating improved outcomes when parents take an active role in the recovery process [100]. In addition to psychotherapy, pharmacotherapy plays an increasingly important role, especially in cases where EDs co-occur with other psychiatric conditions, such as depression or anxiety [66,101]. Studies indicate that SSRIs can help alleviate symptoms of depression and anxiety that often accompany EDs [102]. However, their use in treating the core symptoms of EDs remains less clear. However, their effectiveness in addressing core ED symptoms, such as restrictive eating and binge-eating behaviors, remains unclear. Furthermore, SSRIs and other medications must be prescribed with caution due to potential side effects and age-related considerations.

Nutritional rehabilitation is also a fundamental component of ED treatment [76]. Supervised refeeding strategies, metabolic monitoring, and dietitian involvement help ensure proper weight restoration and prevent medical complications [103]. A multidisciplinary approach that integrates medical, psychological, and nutritional interventions is crucial for achieving long-term recovery.

Moreover, emerging digital health tools, including mobile apps and telemedicine interventions, have shown promise in expanding access to care, particularly for adolescents in underserved areas [104,105]. Digital CBT interventions may be a viable alternative for adolescents who cannot attend in-person therapy, offering a flexible and potentially less stigmatizing treatment option [106]. However, these approaches also present challenges, such as lower adherence rates, limited personalization, and concerns about data privacy. Addressing these limitations is necessary to optimize the effectiveness of digital interventions.

The clinical management of eating disorders in children and adolescents continues to evolve, with an emphasis on early identification, multidisciplinary care, and individualized treatment. Combining psychological therapy, medical monitoring, and family involvement proves to be effective in improving outcomes for eating-disordered pediatric patients. Additionally, preventive strategies, such as school-based programs, social media literacy initiatives, and early screening efforts, may help mitigate ED risk factors before they fully develop. Research into innovative treatment models, such as digital health tools and pharmacological interventions, should refine therapeutic practices and enhance care accessibility for young patients. This progress should also focus on understanding the unique developmental, psychological, and biological factors that contribute to EDs in children and adolescents to advance both prevention and treatment strategies in this vulnerable population.

Meanwhile, BN and BED, both characterized by recurrent episodes of binge eating, may not be immediately recognized in adolescents due to the absence of compensatory behaviors such as purging, which are often seen in adult presentations [21,107]. Furthermore, ARFID, characterized by avoidance of food due to sensory sensitivities or lack of interest, may not be recognized as an eating disorder by clinicians unfamiliar with its manifestation in younger populations [108]. Unlike AN and BN, ARFID is not primarily driven by body image concerns but rather by sensory sensitivities, fear of choking, or lack of interest in eating. Therefore, a nuanced and age-appropriate approach to diagnosis and treatment is essential.

Practical assessment and treatment of EDs in children and adolescents require a comprehensive evaluation. Several studies emphasize the importance of integrating psychological, physical, and family-based assessments in the diagnostic process. Psychological tools, such as the *EDE* and its child-specific version, the *ChEDE* [48,109], are key in identifying disordered eating behaviors, body image disturbances, and cognitive distortions that often accompany EDs. Medical evaluations are equally critical, as eating disorders in children can lead to significant physical health consequences, including stunted growth, delayed puberty, and cardiovascular complications [59]. For example, studies have shown that adolescents with AN often experience delayed bone growth and changes in hormonal regulation that can have lasting effects on their physical development [110,111]. Moreover, family involvement in the assessment process has improved treatment outcomes, particularly in adolescents with AN [112]. FBT has shown significant success, not only in AN but also in other EDs such as BN, where it can help reduce binge-eating episodes and compensatory behaviors [113]. Studies indicate that the efficacy of FBT in treating adolescent EDs is crucial for weight restoration and promoting healthier family dynamics supporting recovery.

## 7. Challenges and Future Directions

While the treatment of eating disorders in children and adolescents has advanced considerably in recent years, significant challenges persist—particularly in early identification, integration of care, and addressing the complex biopsychosocial factors contributing to these conditions. The growing body of research and clinical expertise has led to the development of more refined diagnostic tools and evidence-based interventions; however, many adolescents still go undiagnosed or are misdiagnosed. Early intervention is crucial for successful treatment, but barriers such as stigma, lack of awareness, and the subtlety of symptoms often lead to delays in diagnosis [17,34]. These challenges underscore the need for increased education among clinicians, families, and the general public about the warning signs of eating disorders in children and adolescents.

### 7.1. Early Identification and Misdiagnosis

One of the most significant challenges in the early treatment of pediatric eating disorders is the difficulty in identifying these disorders in their early stages. Symptoms in adolescents may not always be obvious, as eating disorders often manifest in subtle ways, such as an obsession with healthy eating, minor fluctuations in weight, or a gradual decline in social and academic functioning [114]. Despite high prevalence rates of eating disorders, limited screening practices, inadequate training among healthcare professionals, and barriers to help-seeking contribute to persistent low detection rates and significant unmet treatment needs. Key challenges include difficulties in identifying specific types of eating disorders, such as BED and other specified eating disorders, and inadequate diagnostic skills, particularly in healthcare settings [115]. This can also lead to misdiagnoses, as symptoms of eating disorders often overlap with other psychiatric conditions such as depression, anxiety, or OCD [41]. The subtlety of symptoms and the lack of awareness among both clinicians and patients contribute to delays in treatment, which can lead to worse outcomes, including the development of chronic illness and comorbid medical conditions.

Addressing these challenges also requires robust training programs for healthcare professionals, focusing on the subtle signs of eating disorders in adolescents. Training should include recognizing eating disorders in their early stages, understanding the unique presentations in different age groups, and integrating screening tools into routine clinical assessments. Such programs would help clinicians to detect EDs early, thus facilitating timely interventions.

### 7.2. Digital Health Tools for Early Identification

Recent advancements in digital health have opened up new possibilities for the early detection of eating disorders in adolescents. Mobile applications and digital platforms designed for self-monitoring of eating behaviors, mood, and related mental health symptoms can provide valuable insights into the early stages of disordered eating. These tools allow adolescents to track their eating habits, emotional fluctuations, and behaviors associated with body image, enabling early identification of potential issues before they become entrenched [116,117].

For example, some apps are designed to detect early signs of problematic eating behaviors by assessing patterns over time using self-reported data [118]. By tracking daily food intake, exercise habits, and emotional states, these apps can flag unusual patterns that may suggest the onset of an eating disorder. Additionally, some platforms incorporate machine learning algorithms to analyze user data and provide personalized feedback, offering early alerts when a deviation from normal behaviors is detected [119].

These tools empower adolescents to take a more active role in their health and assist clinicians in monitoring progress and detecting warning signs earlier. Clinicians can use the data generated from these digital tools to facilitate timely interventions, even in cases where symptoms are subtle or not immediately apparent. As a result, digital health tools have the potential to reduce delays in diagnosis and improve outcomes by enabling faster, more accurate identification of eating disorders. While these digital tools show promise, they must be integrated into a comprehensive care model with professional clinical support. Further research is needed to validate their effectiveness for early diagnosis and explore their role in enhancing traditional screening methods.

### 7.3. Integration of Care

Another pressing issue in the treatment of pediatric eating disorders is the need for greater integration between pediatricians, mental health professionals, nutritionists, and other specialists. Eating disorders in children and adolescents affect multiple aspects of health—psychological, medical, and nutritional—and therefore require a multidisciplinary approach to treatment [6,7,8,34]. However, treatment often remains siloed, with different providers addressing separate aspects of the disorder without sufficient communication or collaboration. For example, while a pediatrician may focus on the physical health of the adolescent, such as monitoring weight and addressing medical complications, mental health professionals are typically responsible for addressing the psychological factors underpinning the disorder, such as distorted body image and disordered thinking [120]. Similarly, nutritionists play a critical role in guiding the reintroduction of healthy eating behaviors and managing malnutrition. The lack of coordination between these disciplines can lead to fragmented care, failing to address the full complexity of the disorder. Evidence suggests that coordinated, team-based care, in which each professional communicates and collaborates closely with others, leads to better outcomes for adolescents with eating disorders [121,122]. Multidisciplinary care models, which include integrated medical, nutritional, and psychological interventions, should become more effective in achieving long-term recovery and reducing the risk of relapse [123]. Thus, improving care integration is a key priority for improving the management of pediatric eating disorders. Table 5 summarizes the challenges in children and adolescent eating disorder treatment.

## 8. Future Research and Refining Treatment Protocols

Looking forward, further research into the complex biopsychosocial factors—genetic, psychological, and environmental—that contribute to the development of eating disorders in children and adolescents will be crucial for refining treatment protocols and developing more targeted interventions. Studies have highlighted several risk factors for EDs in this age group, including genetic predisposition, family history of mental health issues, societal pressures, and personality traits such as perfectionism [124]. This complex interplay of genetic, psychological, and environmental factors necessitates an integrated treatment approach that holistically addresses the individual. For example, recent work has shown that eating disorders are influenced by genetic, social, and familial factors, with the SLC6A4 gene, which codes for the serotonin transporter, playing a key role. Variants in this gene, particularly the 5-HTTLPR polymorphism, have been linked to ED risk and related psychiatric comorbidities [125]. Future research should explore genetic variants across diverse populations. Understanding these genetic and neurobiological underpinnings could lead to more personalized treatment approaches, potentially allowing for earlier intervention and more effective therapies tailored to the individual’s needs. Moreover, research into the environmental and sociocultural factors—such as peer pressure, media exposure, and cultural beauty standards—also plays a critical role in shaping the development of eating disorders. The increasing recognition of the impact of gender, socioeconomic status, and cultural diversity on the expression of eating disorders will also help in developing more inclusive and effective treatment protocols [126].

Moreover, the influence of social media and digital platforms on adolescents’ body image and self-esteem warrants more research. Studies have shown that exposure to idealized images on social media can exacerbate body dissatisfaction and contribute to disordered eating behaviors, particularly among teenage girls and boys who may feel pressure to conform to unattainable beauty standards [127,128]. The role of digital media as both a risk and protective factor should be explored, offering new avenues for early interventions that target online content consumption as a preventive measure.

In addition to genetic and environmental factors, psychological mechanisms such as cognitive distortions (e.g., all-or-nothing thinking about food and weight), emotion regulation difficulties, and trauma history play an essential role in the development of eating disorders [124]. Future research should focus on how these psychological factors interact with biological predispositions and environmental stressors to create vulnerability to eating disorders. Understanding these mechanisms will help refine psychological interventions and contribute to personalized care strategies.

For instance, recent literature highlights that eating disorders in adolescent boys and young men often involve muscularity-focused concerns, and treatment may need to be adapted to address unique male-specific issues, with athletes and minority groups at elevated risk [129]. Likewise, adolescent populations from marginalized communities, including racial and ethnic minorities, may have unique needs and cultural considerations that must be addressed to ensure they receive adequate care [130].

Integrating diverse cultural, socioeconomic, and gender-related factors into treatment protocols will be essential in providing more individualized and effective care. Clinicians must be aware of cultural perceptions of body image, which may differ across ethnic and cultural groups, and understand how these perceptions influence attitudes toward food, eating habits, and health. For example, in some cultures, eating disorders may be less about weight and more about food-related rituals or control. Thus, developing culturally adapted interventions that acknowledge these unique cultural considerations will improve treatment engagement, adherence, and outcomes.

In summary, advancing our understanding of the genetic, psychological, and sociocultural factors influencing eating disorders in adolescents will be crucial for developing more targeted, inclusive, and effective treatments. Tailoring interventions to individual needs, including considerations for cultural, gender, and socioeconomic diversity, will be essential in improving outcomes and ensuring equitable access to care for all adolescents.

## 9. Conclusions

The treatment landscape for eating disorders in children and adolescents has made significant strides in recent years, with advancements in diagnostic tools, therapeutic interventions, and the growing recognition of the importance of a multidisciplinary approach. However, challenges remain, particularly regarding early identification, the integration of care, and the need for more culturally sensitive treatment protocols. As the field continues to evolve, research into the biopsychosocial factors underlying eating disorders will be crucial in refining treatment approaches, making them more individualized and effective. Early intervention and a comprehensive, family-centered approach will continue to be the cornerstone of successful treatment, offering hope for improved long-term health outcomes for adolescents affected by these severe and complex disorders.

## Figures and Tables

**Table 5:** Challenges and future directions in children and adolescent eating disorder treatment.

Challenges and Future Directions	Key Issues	Potential Solutions
Early Identification and Misdiagnosis	- Subtle symptoms (e.g., minor weight fluctuations, social decline) - Overlapping symptoms with other psychiatric conditions (e.g., depression, OCD) - Limited screening and inadequate training of healthcare professionals - Barriers to help-seeking due to stigma and lack of awareness	- Enhanced training for clinicians on subtle ED presentations - Routine screening tools integrated into clinical settings - Increased public and family education on ED warning signs
Digital Health Tools for Early Identification	- Limited early detection methods in traditional settings - Need for tools to track eating behaviors and emotional patterns - Lack of research on effectiveness of digital tools	- Development of apps for self-monitoring of eating habits and mood - AI-driven tools for personalized feedback and early alerts - Integration of digital tools into clinical practice with professional oversight
Integration of Care	- Siloed treatment among pediatricians, mental health professionals, and nutritionists - Lack of communication leading to fragmented care - Need for multidisciplinary approaches	- Adoption of team-based, integrated care models - Improved communication among healthcare providers - Holistic treatment plans addressing medical, nutritional, and psychological needs
